# Pharmaceutical Regulation in Central and Eastern European Countries: A Current Review

**DOI:** 10.3389/fphar.2017.00892

**Published:** 2017-12-18

**Authors:** Paweł Kawalec, Tomas Tesar, Lenka Vostalova, Pero Draganic, Manoela Manova, Alexandra Savova, Guenka Petrova, Zinta Rugaja, Agnes Männik, Christoph Sowada, Ewa Stawowczyk, Andras Harsanyi, Andras Inotai, Adina Turcu-Stiolica, Jolanta Gulbinovič, Andrzej Pilc

**Affiliations:** ^1^Health Sciences Faculty, Institute of Public Health, Jagiellonian University Medical College, Kraków, Poland; ^2^Department of Organization and Management in Pharmacy, Faculty of Pharmacy, Comenius University in Bratislava, Bratislava, Slovakia; ^3^Health Technology Assessment Department, Pricing and Reimbursement Regulation Branch, State Institute for Drug Control, Prague, Czechia; ^4^Croatian Agency for Medicinal Products and Medical Devices, Zagreb, Croatia; ^5^Faculty of Pharmacy, Medical University of Sofia, Sofia, Bulgaria; ^6^The National Health Service, Ministry of Health, Riga, Latvia; ^7^Institute of Family Medicine and Public Health, University of Tartu, Tartu, Estonia; ^8^National Institute of Health Insurance Fund Management, Budapest, Hungary; ^9^Department of Health Policy and Health Economics, Eötvös Loránd University, Budapest, Hungary; ^10^Syreon Research Institute, Budapest, Hungary; ^11^Faculty of Pharmacy, University of Medicine and Pharmacy of Craiova, Craiova, Romania; ^12^Departament of Pathology, Forensic Medicine and Pharmacology, Vilnius University, Vilnius, Lithuania; ^13^State Medicine Control Agency, Vilnius, Lithuania; ^14^Departament of Neurobiology, Institute of Pharmacology, Polish Academy of Sciences, Krakow, Poland

**Keywords:** pricing, reimbursement, CEE, pharmaceutical regulation, drug policy

## Abstract

**Objectives:** The aim of this study was to review reimbursement environment as well as pricing and reimbursement requirements for drugs in selected Central and Eastern Europe (CEE) countries.

**Methods:** A questionnaire-based survey was performed in the period from November 2016 to March 2017 among experts involved in reimbursement matters from CEE countries: Bulgaria, Croatia, Czech Republic, Estonia, Hungary, Latvia, Lithuania, Poland, Slovakia, and Romania. A review of requirements for reimbursement and implications of Health Technology Assessment (HTA) was performed to compare the issues in above-mentioned countries. For each specified country, data for reimbursement costs, total pharmaceutical budget, and total public health care budget in the years 2014 and 2015 were also collected. Questionnaires were distributed via emails and feedback data were obtained in the same way. Additional questions, if any, were also submitted to respondents by email. Pricing and reimbursement data were valid for March 2017.

**Results:** The survey revealed that the relation of drug reimbursement costs to total public healthcare spending ranged from 0.12 to 0.21 in the year 2014 and 2015 (median value). It also revealed that pricing criteria for drugs, employed in the CEE countries, were quite similar. External reference pricing as well as internal reference pricing were common in mentioned countries. Positive reimbursement lists were valid in all countries of the CEE region, negative ones were rarely used; reimbursement decisions were regularly revised and updated in the majority of countries. Copayment was common and available levels of reimbursement differed within and between the countries and ranged from 20 to 100%. Risk-sharing schemes were often in use, especially in the case of innovative, expensive drugs. Generic substitution was also possible in all analyzed CEE countries, while some made it mandatory. HTA was carried out in almost all of the considered CEE countries and HTA dossier was obligatory for submitting a pricing and reimbursement application.

**Conclusions:** Pricing and reimbursement requirements are quite similar in the CEE region although some differences were identified. HTA evaluations are commonly used in considered countries.

## Introduction

In the European Union (EU) pricing and reimbursement of pharmaceuticals is primarily a national competence; that is reason for different pharmaceutical regulation systems currently applied in these countries. A review of pharmaceutical pricing and reimbursement systems in the countries of Western Europe was performed in some publications (Vogler et al., 2011; Panteli et al., 2016; Allen et al., 2017). However, an urgent need for review of data on the pharmaceutical systems in the Central and Eastern Europe (CEE) countries as well as a strong interest in pricing and reimbursement strategies applied in these countries is observed.

In the EU, medicines can be placed on the market only if they have received a marketing authorization from the European Commission or from competent national authorities as EU legislation provides harmonized rules of drug authorization^1^. Pharmaceutical therapies are often subsidized by national reimbursement systems in order to provide the adequate access of medicines to citizens, which influences the prescription and utilization of medicines. Member States are free to adopt their own pricing and reimbursement regulations, as long as these are in line with the minimal procedural requirements of Directive 89/105/EEC^2^^,^^3^ issued by the European Commission to ensure the transparency of national pricing and reimbursement regulations (for this reason, it is commonly referred to as the Transparency Directive).

The Transparency Directive requires that decisions related to the reimbursement of drugs have to be taken within 90 days of application (and 180 days for reimbursement and pricing decisions); the directive also provides major requirements with respect to individual pricing and reimbursement decisions which:

- Must be announced to the applicant and have to state of reasons based on verifiable and objective criteria;
- Must be open to appeal in court at national level^3^.

As more specific and detailed requirements in pricing and reimbursement policy are set on national level, we performed a review of currently established drug policies in CEE countries to compare and provide data to fill a significant knowledge gap.

Positive or negative reimbursement lists are in use to provide information on reimbursement or contraindication to reimbursement of specific drugs (Panteli et al., 2016). Medicines present in the Positive Drug List (PDL) are reimbursed at different levels due to partial or full reimbursement. For drugs which are reimbursed at a level lower than 100%, patients' copayment is needed; medicines included in negative lists are not reimbursed in a specific country; reimbursement categories are country specific (Vogler, 2012). For drugs which are expensive and used chronically the reimbursement levels are usually higher than for drugs used temporarily (e.g., antibiotics).

To have a drug reimbursed, marketing authorization holder (MAH) has to submit an application with a number of attachments, including health technology assessment (HTA) dossier (Panteli et al., 2016). HTA is useful for reimbursement decisions for new molecules to assess value for money; it usually includes economic evaluation of cost effectiveness and budget impact analysis (BIA), clinical effectiveness, and safety profile assessment of a particular drug in relation to the therapeutic alternative (Panteli et al., 2016; Zawada and Mäkelä, 2017).

Medicines which are not reimbursed can be financed from public funds within compassionate use programs. This way of public coverage for pharmacotherapy allows for reimbursement of specific medicines for particular patient after approval of individual application (Balasubramanian et al., 2016).

Many European countries applied a reference price system, with a maximum reimbursement level specified for groups of interchangeable pharmaceuticals (Panteli et al., 2016). External reference pricing (also known as international reference pricing or external price referencing) was frequently employed strategy in price regulation comparing the prices of the same product in other countries. External reference pricing is usually applied for reimbursable, original drugs, but vary substantially across countries. Another reference system is internal reference pricing, that is, comparing the price of a product to the prices of similar products in the same country, or in the same therapeutic class, or in the same International Nonproprietary Name (INN) in order to set the reference price as a function of prices of domestic substitutes. Physicians are allowed or even obliged to prescribe INN instead of the trade name. It allows the generic substitution—patient is offered to buy the cheapest or any other product with a particular active substance (Panteli et al., 2016).

For some medicines, the risk-sharing schemes or risk-sharing agreements (RSAs) could be applied (Panteli et al., 2016); based on such agreements MAH is obliged to take the proportion of risk and expenditure resulting from financing of the new pharmacotherapy from public resources.

The country sample was chosen to include all CEE countries which are the “new” EU members, that is, which joined the EU this century: Bulgaria, Croatia, Czech Republic, Estonia, Hungary, Latvia, Lithuania, Poland, Slovakia, and Romania. Croatia is the newest member of the EU, as it joined in 2013, Bulgaria and Romania entered EU in 2007, and the remaining of the included countries joined the EU in 2004^4^.

The main goal of this study was to illustrate in a systematic, comparative manner current requirements and regulations influencing pharmaceutical systems in considered European countries. The investigation included: pricing and price updates criteria, patient copayments, generic substitution, health technology assessment implementation and comprehensive review of reimbursement requirements.

## Materials and methods

We performed country-based reviews that provided a detailed description of pharmaceutical system and policy initiatives which are valid or under development in a specific country; it was carried out in the period from November 2016 to March 2017. Each review was produced by 1 or more leading national experts in pricing and reimbursement policy; the study was supervised by the project coordinator.

Experts from the participating countries provided in-depth data valid for sophisticated assessment of pricing and reimbursement as well as data on expenditures on specified pharmacotherapies. In order to facilitate comparisons between countries, reviews were based on a questionnaire survey, covering all the project objectives; the questionnaire was prepared after consideration of some previously performed studies (Panteli et al., 2016; Kawalec et al., 2017). The first part of our survey included questions referring to general pricing and reimbursement environment in reference countries. Additionally, data on total public healthcare expenditure, total reimbursement costs, market share of generics in the years 2014 and 2015 were provided. In the subsequent parts of our study, more accurate data on pricing and reimbursement policy were provided. Additionally, it included specific reimbursement implications embracing generic substitution, reimbursement control, and reimbursement decision-making process, including HTA dossier requirements.

Questionnaires were distributed via emails and feedback data were obtained in the same way. An interactive review with the contributors was performed to obtain the optimal range of information needed and to get additional answers in case of any lacking data. Pricing and reimbursement data were valid for March 2017.

## Results

### General overview

We reviewed data on total public expenditure (in Euro) on reimbursement of medicines and total public health care budget (in Euro) in the years 2014 and 2015 for considered countries. For several countries—Bulgaria, Croatia, Lithuania, and Romania—data concerned total public healthcare expenditures were not available. It was revealed that the relation of drug reimbursement costs to total public healthcare spending in the year 2014 ranged from 0.12 to 0.21 and the median value was 0.18. For the year 2015 more limited data were available (no data concerned reimbursement costs for Hungary), but the ratio was quite similar and equaled 0.12 to 0.21 and median value was 0.15 (see Table 1 for details). Observed differences in pharmaceutical expenditures should be interpreted in conjunction with the different price levels and volume composition of consumption as well as impact on measurement by dispensation practices.

**Table 1:** Total public expenditure on reimbursement and total public healthcare budget (in thousands Euro; presented for 2014 and 2015).

**Aspect required**	**Bulgaria**	**Croatia**	**Czechia**	**Estonia**	**Hungary^a^^–^^d^**	**Latvia**	**Lithuania^g^**	**Poland^i^**	**Romania**	**Slovakia^e^^,^^f^**
Total public expenditure (in Euro) on reimbursement of drugs in 2014	544,014	667,500	2,089,560	109,753	1,028,000	118,930	190,600	2,453,500^j^	1,838,000	884,000
Total public health care budget (in Euro) in 2014^h^	n/a	n/a	10,261,656	924,727	5,020,590	776,287	n/a	18,382,789	n/a	4,217,012
Public reimbursement and total public health care budget relation in 2014	n/a	n/a	0.20	0.12	0.20	0.15	n/a	0.13	n/a	0.21
Total public expenditure (in Euro) on reimbursement of drugs in 2015	559,353	702,300	2,293,856	112,801	n/a	124,300	197,500	2,558,466	1,774,000	905,000
Total public health care budget (in Euro) in 2015^h^	n/a	n/a	10,751,286	973,900	5,159,609	822,601	n/a	18,853,265	n/a	4,398,045
Public reimbursement and total public health care budget relation in 2015	n/a	n/a	0.21	0.12	n/a	0.15	n/a	0.14	n/a	0.21

*n/a, Not available*.

a *Public pharmaceutical spending in 2014 by the National Health Insurance Fund of Hungary: http://www.neak.gov.hu/felso_menu/szakmai_oldalak/publikus_forgalmi_adatok/gyogyszer_forgalmi_adatok/gyogyszer_forgalmi_adatok_2014.html*.

*^b^Calculated on current exchange rate in 2014 by the Central Bank of Hungary*.

*^c^Public pharmaceutical spending in 2015 by the National Health Insurance Fund of Hungary: http://www.neak.gov.hu/felso_menu/szakmai_oldalak/publikus_forgalmi_adatok/gyogyszer_forgalmi_adatok/gyogyszer_forgalmi_adatok_2015.html*.

d *Calculated on current exchange rate in 2015 by the Central Bank of Hungary*.

e *Slovak Ministry of Finance, http://www.finance.gov.sk/Default.aspx?CatID=11158*.

f *National Health Information Centre (NHIC), Slovak Republic, http://www.nczisk.sk/en/Pages/default.aspx*.

g *Includes only reimbursed medicines for outpatient care; does not include expenses for centrally purchased reimbursed medicines and expenses for medicinal aids*.

h *Organization for Economic Co-operation and Development, Health expenditure and financing, Current expenditure on health, Government schemes and compulsory contributory health care financing schemes, stats.oecd.org*.

i *National Health Fund, spending on reimbursement, www.nfz.gov.pl*.

j *Costs of reimbursement of drugs by the NHF (ambulatory list, catalog of chemotherapy, drug programs); costs of drugs administered in hospitals as a part of DGR procedures were not included*.

We also measured timelines for pricing and reimbursement decisions among the analyzed countries; in majority of them a 180 days deadline for decision-making process, with accordance to the EU Transparency Directive, was applied.

In Hungary, the official timelines depend on the type of a procedure applied: for a standard procedure it is usually 90 days but for a simplified procedure 60 days is enough to issue a reimbursement decision^5^. In Romania, the decision is announced to the applicant within 90 days from the submission of the complete documentation. If the evaluation of requested drug has not been approved in price submission, the time for final decision is extended by next 90 days. In Czech Republic, the decision is made within 75 days in the case of application only for price or only for reimbursement (165 for joint application for price and reimbursement). Since 2012, a special type of short 30-days procedure is available for generic and biosimilar products. In Estonia, the reimbursement decision for the standard (simplified) procedure is made within 90 days from submission, and when the new HTA dossier is submitted, the decision is made within 180 days^6^. In Lithuania preliminary recommendation alongside with assessment report on therapeutic value, pharmacoeconomic value and budget impact is published on the website of the Ministry of Health and informs the Applicant within 80 days from submission of the dossier^7^.

### Review of pricing criteria

In all considered countries an external (international) reference pricing as well as internal reference pricing is common. The number of reference countries differs significantly in case of external price referencing: the lowest number is used in Estonia (3 reference countries^6^) as well as Croatia (5), and the highest numbers were reported for Hungary (31) and Poland (31).

We also analyzed influence of considered countries on others in terms of reference pricing; Cyprus, Iceland, Malta, Luxembourg, and Norway occurred as reference countries quite rarely, while Slovakia, Czech Republic, and Lithuania were used as a reference most often in the analyzed CEE countries (see Tables 2, 3 for detail).

**Table 2:** Pricing policy in analyzed CEE countries.

**Aspect required**	**Bulgaria**	**Croatia**	**Czechia**	**Estonia**	**Hungary**	**Latvia**	**Lithuania**	**Poland**	**Romania**	**Slovakia**
Is external (international) reference pricing obligatory?	Yes	Yes	Yes	Yes	Yes	Yes	Yes	Yes	Yes	Yes
Number of reference countries considered while external reference pricing	17	5	23	3	31	7	8	31	27	28
Is internal reference pricing obligatory?	Yes	Yes	Yes	Yes	Yes	Yes	Yes	Yes	Yes	Yes

**Table 3:** Reference countries considered while external reference pricing.

**Country**	**Bulgaria**	**Croatia**	**Czechia**	**Estonia**	**Hungary**	**Latvia**	**Lithuania**	**Poland**	**Romania**	**Slovakia**	**Number of referencing countries^∧^**
Austria											4
Belgium											6
Bulgaria											5
Cyprus											4
Czechia											8
Croatia											5
Denmark											7
Estonia											7
Finland											6
France											7
Germany											4
Great Britain											5
Greece											6
Hungary											7
Iceland											4
Ireland											4
Italy											7
Latvia											8
Liechtenstein											4
Lithuania											8
Luxembourg											4
Malta											4
Netherlands											5
Norway											3
Poland											6
Portugal											6
Romania											6
Slovakia											9
Slovenia											6
Spain											7
Sweden											5
Switzerland											3

∧ *number of countries which refer to the particular one. Reference countries are marked in green*.

In the case of Bulgaria, for products containing the same INN which were in the same pharmaceutical form the value of reimbursement is set at the level of the cheapest product determined by the cost per defined daily dose^8^. The ex-factory price of the generic version of a medicine listed in the PDL may not exceed 70% of the ex-factory price of the reference product in the PDL. The generic pricing is also subject to external referencing, so the established ex-factory price cannot be higher than the lowest manufacturer price in the reference countries.

In Croatia, the basis for determining the comparative wholesale price of the drug is the wholesale price of the same drug (a drug with the same generic name and the same pharmaceutical form) in Italy, Slovenia, and the Czech Republic, and, if necessary, in Spain and France. The Croatian Health Insurance Fund (CHIF) established standards and price settings for services covered by the Fund and it is responsible for pricing and the reimbursement decisions on drugs and medical devices which are included in the CHIF list of drugs by the experts' council of the CHIF—Committee for Medicines^9^^,^^10^.

For Czech Republic, reference countries are all countries of the EU except Austria, Bulgaria, Cyprus, Estonia, Germany, Luxembourg, Malta, and Romania. External price referencing embraces at least 3 prices. Exclusion of the price extremes compared to other reference countries is applied^11^. The lowest price is considered a price extreme if it is lower by more than 20% of the average of 2nd and 3rd lowest price found in other reference countries. If there is no price extreme identified, ex-factory price should be equaled to an average of 3 lowest prices. In the case of a price extreme, the lowest price is excluded and ex-factory price is set on the level of an average of 2nd and 3rd lowest prices, or based on a price agreement concluded between the public payer and MAH if it would lead to a lower price^18^.

If a pharmaceutical (with the exception of highly innovative drugs) is not on the market in at least 3 reference basket states, price agreement can be used. If none of the above-mentioned procedures is applicable, the price is set as the maximum ex-factory price of the closest therapeutically comparable pharmaceutical available in the Czech Republic or in the reference basket countries. For highly innovative drugs, it is possible to set the ex-factory price as the average manufacturer's price revealed in at least 2 reference basket states.

Currently, in Czech Republic the price and reimbursement of the first generic product has to be at least 40% lower than the price and reimbursement of the reference drug. In the case of other generic product the only the price is decreased. Similarly, in the case of biological products, the price and reimbursement have to be at least 30% lower.

In Estonia, the price of especially expensive drugs is usually negotiated between the Ministry of Social Affairs, Health Insurance Fund, and the pharmaceutical company. The main measures are external price referencing and price comparisons with other similar medicines. Thus, both external and internal pricing is applied but also the following criteria are used:

- a generic needs to be 30% cheaper than the originator in the same group,
- a biosimilar needs to be 15% cheaper than the originator in the same group,
- a parallel imported medicine needs to be 10% cheaper than the originator in the same group,
- in a reference group, the first 3 medicines with the same active substance and the same route of administration should be 10% cheaper from each other and the next medicines to be included into this group should not be more expensive than the cheapest medicine in this group (Regulation of Ministry of Social Affairs, 2004).

Manufacturers in Hungary are free to determine the manufacturing price of their pharmaceutical products; however, they may be forced to decrease the proposed price during the procedure for inclusion of a product into reimbursement. External and internal price referencing is applied for reimbursed innovative and off-patent medicines, respectively. In case of external price referencing, all EU member states are the reference countries^12^, the lowest price within the basket of countries is defined as the reference price. In internal price referencing, the lowest price drug is the reference product, with a predefined percent of reimbursement. Drugs with a slightly higher price receive the same amount of reimbursement but with a higher copayment. Drugs with significantly higher price are delisted from reimbursement. From 2011, a blind bidding procedure is used to set the generic reference price. Every 6 months, generic manufacturers may submit their price reduction proposals, but without knowing the proposals submitted by their competitors. After the closure of the bidding process, the drug with the lowest price becomes the reference product. Products with less than 15% price differential have equal amount of reimbursement with the reference product, while products with more than 15% price difference compared to the reference product can have only 85% of the reimbursement given to the reference product.

In Latvia, the price should not exceed the prices used in Estonia and Lithuania, and the third lowest price in Czech Republic, Romania, Slovakia, Hungary, and Denmark. All drugs with the same indication are reimbursed at the same rate. In the case of pharmaceuticals with calculated reference price, the reimbursement rate is applied to the reference price. The products are grouped into clusters within the INN or within the pharmacotherapeutic group if there are no clinically relevant differences in the efficacy and safety profile for the same indication and drugs are intended for the same patients' group; the products are clustered according to the dosage and pharmaceutical form. Then the reference product for each cluster is identified (the cheapest pharmaceutical), and on the basis of the price of the reference product the reimbursement price for each pharmaceutical in the cluster is calculated.

In Lithuania, the declared price of the first generic in a group should be 50% lower than the price of the original product, price of the second generic must be at least – 15% lower than the first generic, and the third generic–15% lower than the second one. Declared price of biosimilars should be at least 30% lower than that of original product^13^.

In Poland, external reference pricing and internal reference pricing (reimbursement limit is set for each cluster) are valid, but also value-based pricing (somehow linked to threshold prices in the economic analysis) is under consideration and influences the price of a drug. The maximum price of generic product cannot exceed 75% of the only drug with this active substance. After evaluation performed by the Polish HTA Agency (pol. *Agencja Oceny Technologii Medycznych i Taryfikacji*), negotiations with the Economic Committee in the Ministry of Health are crucial.

In Romania, Emergency Ordinance no. 59/28.09.2016^14^ established the methodology regarding the calculation, procedure for endorsement and approval of the maximum prices of medicinal products licensed on the Romanian pharmaceutical market, except medicines obtained without a prescription (over-the-counter)^15^. The manufacturer's price should be lower than or equal to the price of the same product from the list of reference countries. If the drug does not have an established price in any of 12 countries, it must be lower than or equal to the price of the same drug in the country of origin. The reference price of the generic is 65% compared to the producer price of innovative drug. Generic reference price remains unchanged, despite the change in price of innovative drug. The biosimilar reference price represents 80% of the producer price of the reference biologic drug^14^^,^^15^. Updating the reference prices for generics and biosimilars is done annually in October by applying the latest average exchange rate (set for third quarter) of RON to EUR by the Romanian National Bank.

Slovakia has implemented a reference pricing system for medicines, and a maximum price is set for a standard daily dose in each specific reference group of medicines based on that system. All medicines included in such reference group [medicines with the same 5-digit Anatomical Therapeutic Chemical (ATC) classification system code] contain the same active substance per dose and are administered in the same form. Besides, internal reference pricing is applied to some therapeutic groups which form internal reference groups of drugs that have the same molecular structure (medicines with the same 4-digit ATC code). Likewise, a maximum price for a standard daily dose of drugs belonging to the same internal reference group is defined. As a result, prices of pharmaceuticals with different active substances are linked to the cheapest alternative within an internal reference group. Changes in price for a particular medicine may thus influence the reimbursement of other medicines in the same internal or external reference group. The maximum price of the first generic product cannot exceed 65% of the original drug with this active substance, and the maximum price of the first biosimilar product cannot exceed 80% of the biological drug with this active substance^16^.

### A review of reimbursement policies in considered CEE countries

In considered countries, diversified legislation and requirements concerned reimbursement policy are in use. Details on those issues were presented in Tables 4–6.

**Table 4:** Reimbursement policy review in analyzed CEE countries.

**Aspect required**	**Bulgaria**	**Croatia**	**Czechia**	**Estonia**	**Hungary**	**Latvia**	**Lithuania**	**Poland**	**Romania**	**Slovakia**
Is there a positive reimbursement list available?	Yes	Yes	Yes	Yes	Yes	Yes	Yes	Yes	Yes	Yes
Is there a negative reimbursement list available?	No	No	No	No	Yes	No	No	No	Yes	No
Are there co-payments for pharmaceuticals?	Yes	Yes	Yes	Yes	Yes	Yes	Yes	Yes	Yes	Yes
How often are reimbursement decisions revised (periodic/*ad hoc* revisions)?	Once a year	Periodic several times a year	Periodic once a 5 years but also *ad hoc* revision is possible	Periodic every 3 months	*ad hoc* revision	Periodic, every 3 months	*ad hoc* revision	Periodic, every 2 months	1 year or *ad hoc* revision	Periodic, every 3 months
Are risk sharing agreements (RSA) available in reimbursement decision making?	Yes	Yes	Yes	Yes	Yes	Yes	Yes	Yes	Yes	Yes
Is a cap for patients for reimbursement expenses?	Yes, currently in just some cases	No	No	Yes	No	No	No	No	Yes	Yes, but only for vulnerable groups of patients

**Table 5:** Reimbursement levels in considered CEE countries.

	**Bulgaria**	**Croatia**	**Czechia**	**Estonia**	**Hungary**	**Latvia**	**Lithuania**	**Poland**	**Romania**	**Slovakia**
Level of reimbursement	100, 75, 50, 25%	100 or 80%	There are no levels or categories of co-payments	100, 90, 75, 50%	Normative reimbursement: 25, 55, 80% Indication-linked reimbursement: 50, 70, 90, 100%	100, 75, 50%	100, 90, 80, 50% -list A 100, 50% - list B 100% - list C	100, 70, 50% Flat price co-payment	90% - list A 50, 90% - list B 100% - list C 20% - list D	100% or partial (between 0 and 100%)

**Table 6:** Reimbursement specific implications in considered CEE countries.

**Aspect required**	**Bulgaria**	**Croatia**	**Czechia**	**Estonia**	**Hungary**	**Latvia**	**Lithuania**	**Poland**	**Romania**	**Slovakia**
Is INN prescribing possible/mandatory?	Possible	Possible	Possible	Mandatory	Possible (as a pilot for statins)	Possible	Mandatory	Possible	Mandatory with some exceptions (biologic drugs, for example)	Mandatory for selected substances; prohibited for the others
Is a generic substitution possible or mandatory (obligatory)?	Possible	Possible	Possible	Mandatory	Mandatory	Possible	Possible	Possible	Possible with some exceptions (biologic drugs, for example)	Possible
Are there any special reimbursement regulations (e.g., easier availability for patients) for orphan drugs?	No	No	Possible	No	No	No	Yes	No	No	Yes, only for prevalence lower than 1:100,000
Are drugs for “compassionate use” reimbursed?	No	No	Yes	n/a	Yes	No	No	No	No	No
Are prescribing guidelines used for reimbursed drugs?	Yes	Yes	Yes dependant mainly on reimbursement condition which correspond to the different range of SPC (narrower or broader indications)	Yes	Yes	Yes	Yes	No	Yes	Yes
Is electronic prescribing available/planned?	Planned/in construction	Yes	Planned/in construction	Yes	Planned/in construction	Yes	Yes	Planned/in construction	Yes	Planned/in construction
In case of reimbursed drugs there is a physicians/prescribers prescription monitoring?	Yes	Yes	No	Yes	Yes	Yes, in some cases	Yes	Yes	Yes	Yes
In case of reimbursed drugs there are pharmaceutical budgets, volume caps for physicians/prescribers?	No	No	Yes	No	Yes	No	No	No	Yes	No

*INN, International Nonproprietary Name*.

In Bulgaria, a Positive Drug List (PDL) is essentially used; it consists of 4 appendixes:

- Medicines paid by the National Health Insurance Fund for outpatients,
- Medicines paid from the hospital budget for inpatients,
- Medicines paid by the budget of the Ministry of Health—medicines for the diseases which are out of the scope of Health Insurance, e.g., tuberculosis, HIV/AIDS, as well as vaccines for obligatory immunization,
- prices of all medicines in PDL including: manufacturer price, wholesale and retail margins expressed as value and percentage, value added tax.

Copayment is valid only in case of drugs listed in appendix 1, for outpatient care. The reimbursement levels are as follows:

- 100% for the medicines indicated in the therapy of chronic diseases, leading to severe disruptions in the quality of life or disablement and requiring prolonged treatment,
- 75% for the medicines for diseases with a chronic course and widespread prevalence,
- 25 or 50% for the medicinal products for the diseases other than those mentioned above.

The reimbursement level depends on the type of the disease, the type of a treatment (essential, symptomatic, palliative, etc.), clinical significance, and budget resources allocated for procurement of the medicine. The copayment vary depending on the disease (socially important diseases and rare diseases have higher reimbursement level) and the results of product's assessment (efficacy/therapeutic effectiveness, safety, and pharmacoeconomic criteria).

In Croatia, there are two positive reimbursement lists: the essential (basic) list of medicines, and the supplementary list of medicines^9^^,^^10^. The CHIF covers costs of a drug from a supplementary list at the price of an equivalent drug specified under the essential list. The rest of the amount is covered by an individual copayment. Copayments are required only for pharmaceuticals which are on the supplementary list of drugs. The essential (basic) list of medicines contains medications clinically and economically most appropriate for the treatment of diseases. The official price of reimbursed drug is established in the public bidding procedure according to special regulations. The supplementary list of medicines contains drugs with a higher level of prices compared to the prices of drugs from the essential list. The cost of drugs from the supplementary list is covered by CHIF at the price specified under a special law of an equivalent drug from the essential list of medicines and additional cost has to be covered by patient as a copayment.

Copayment does not vary depending on product, patient, or characteristics of the disease, and it is the same for all drugs. The drugs are included in the list of drugs through the expert council of the CHIF and the process is transparent.

In Czech Republic, there is a limit for yearly copayment of 5000 CZK (approx. 190 EUR) for children up to 18 years of age and 2500 CZK (approx. 95 EUR) for seniors over 65 years of age (i.e., cap for supplementary payment). According to legislation (Act. No. 48/1997), the State Institute for Drug Control (Czech, Státní ústav pro kontrolu léčiv, SUKL) once in the course of 5 years is obligated to make a complex review of reimbursement level and prices of all pharmaceuticals which belong to specific groups or therapeutic clusters of pharmaceuticals (so-called reference groups). These reference groups are formed by pharmaceutical products which have similar efficacy, safety, and position in clinical practice. Through these complex reviews, the level and conditions for reimbursement of all pharmaceutical products are re-evaluated in relation to evidence based medicine and valid clinical guidelines. SUKL also carries out simple short-term reviews initiated when the first generics enter the market or if a reduction of the price of a particular drug can create substantial savings in a whole reference group. SUKL is also obligated to assure that at least 1 product in each therapeutic group which are defined in Act. No. 48/1997 is fully reimbursed. Public payers can also initiate the simple short-term reviews of the reference groups if they expect substantial savings^17^^,^^18^.

In Estonia, patients' copayments for reimbursed drugs are as follows:

- 1.27 EUR for medicines reimbursed at 100% level,
- 1.27 EUR and additionally 10% of the price exceeding the agreement price for medicines reimbursed at 90% level,
- 1.27 EUR and additionally 25% of the price exceeding the agreement price for medicines reimbursed at 75% level,
- 3.19 EUR and additionally 50% of the price exceeding the agreement price for medicines reimbursed at 50% level^19^.

The reimbursement rate depends on the severity of condition or specific disease. This means that there is a list of diseases established by the Ministry of Social Affairs for which the medicines need to be reimbursed with specific percentage level^20^. This arrangement is similar to that in Latvia.

In Hungary, the reimbursement levels are established according to the specifications of the therapies; the more severe and chronic the disease, the higher the reimbursement level is.

In the case of outpatient setting:

- normative reimbursement applies to all physicians with general prescription rights and may be used for all indications listed in the Summary of Product Characteristics (SPC); this technique is associated with a reimbursement level of 25, 55, and 80%, resulting in a level of patient's copayment of 75, 45, and 20%, respectively;
- indication-linked reimbursement restricts reimbursement to only certain professionals who can prescribe the drug; the reimbursement is granted only in a subgroup of authorized indications and is granted if the conditions defined by the payer are met; the level of reimbursement is 50, 70, 90, and 100%, resulting in a level of patient's copayment of 50, 30, 10, or 0%; for each unit, there is a fixed prescription fee of 300 HUF (1 EUR is approximately 315 HUF [2016]) for drugs with 0% copayment.

For off-patent products, the lowest price of a drug is the reference price which has a predefined percentage of reimbursement. Medicines with slightly higher price receive the same amount of reimbursement, but with higher copayment. Drugs with significantly higher price are delisted from reimbursement. In the case of inpatient setting, there is no copayment.

In Latvia, medicinal products are reimbursed depending on the diagnosis; reimbursement categories are set according to the severity of the disease and are as follows:

- 100% reimbursement – for chronic, life threatening diseases or diseases causing irreversible disability, where the use of pharmaceuticals ensures and maintains the patient's life functions,
- 75% reimbursement – for chronic diseases, for which the maintenance of the patient's life functions are aggravated without the use of pharmaceuticals,
- 50% reimbursement – for vaccines and for diseases where pharmaceuticals maintain or improve patient's health.

Patients have to pay the copayment in the case of the 75 and 50% reimbursement levels^21^.

In Lithuania, the following reimbursement levels are applied:

- 100, 90, 80, 50% of reimbursed price according to list A (the list of diseases and medicines); the level of reimbursement depends on the severity, disablement and chronic course of disease,
- 100, 50% of reimbursed price according to list B (the list of medicines for certain social groups); 100% reimbursement for children, 50% for pensioners,
- 100% of reimbursed price for medicinal aid for outpatient care according to list C.

- The following reimbursement levels are employed in Poland:
- 50% and costs above the reimbursement limit,
- 30% and costs above the reimbursement limit,
- flat price over reimbursement limit,
- free of charge (e.g., drug programs for oncology patients, chemotherapy, patients older than 75 years, war-disabled).

The reimbursement levels depend on the type and duration of the disease and treatment costs (Kawalec and Malinowski, 2016; Jahnz-Rózyk et al., 2017). Drugs used in inpatient care are fully covered from public funds via drug programs or catalog of chemotherapy. They can also be reimbursed indirectly within hospitalizations (Diagnosis-related Groups) or medical procedures carried out in hospitals.

In Romania, there are copayments for pharmaceuticals. There are 4 reimbursement lists: list A (reimbursement rate 90%), list B (50%), list C (100%) divided into sub-lists C1, C2, and C3, and list D (20%). Even if a drug is on list C, copayments could be required, and even if a drug is on list A (90%), copayments do not have to be required. If a drug from the list B is prescribed for a pensioner with small pension, it becomes 90% compensated. Copayments vary based on patient characteristic (age, income) or condition (chronic or acute/subacute).

In Slovakia, there are the following reimbursement levels: 100% or partial reimbursement (between 0 and 100%). The decision on reimbursement levels for medicines eligible for partial reimbursement is based on the following criteria: therapeutic benefit of the medicine, its final retail price (cost-effectiveness), and the prices of other reimbursed medicines within the same reference group^16^.

There is always at least 1 medicine available in a determined therapeutic class with no copayment. Copayments do not vary based on indication, effectiveness, or innovation, but they can vary based on age, income, characteristics, or condition of disease. There are drugs in Slovakia which are fully covered for vulnerable people (e.g., disabled people, retired people, children)^16^.

### Specific measures for the assessment of medicines, particularly costly pharmaceuticals

In Bulgaria, there are obligatory discounts for all costly medicines, but they are confidential between the MAH and the public fund. For medicines paid by hospitals, public tenders are employed. RSAs, usually in a form of price volume agreements, are also used and they constitute one of the conditions for inclusion in the PDL. In some cases, a cap for patients is used as part of the RSA.

In Croatia, there are specific measures for assessment of drugs such as value-based pricing, rebates, and public tendering. RSAs are available especially for the biological or biosimilar drugs.

In Czech Republic highly innovative drugs can get temporary reimbursement for up to 3 years even without relevant cost-effectiveness and BIA, which means an accelerated entry to the market. However, after 3 years of temporary reimbursement, they have to meet cost-effectiveness and budget impact criteria to get permanent reimbursement. Health care funds can also cover the cost of a product which is not reimbursed (usually not in a particular off-label indication), based on individual application filed for a specific patient by a physician.

RSAs or cap budget limits agreed between MAH and payers can be a necessity for coping with the required HTA parameters, especially in the case of new, costly molecules. However, no agreements (budget cap, RSA) are legally required as a condition for reimbursement. Legally, there is no formal cap for reimbursement for an individual patient.

In Estonia RSAs are rare, because they constitute a high administrative burden to the Health Insurance Fund. The price of costly medicines is usually negotiated between the Ministry of Social Affairs, Health Insurance Fund and the Marketing Authorization Holder. Additional reimbursement from Health Insurance Fund is possible when patient's payments per prescription medicines exceeds 300 EUR per year.

In Hungary, managed entry agreements are mandatory conditions for reimbursing any new drugs, and obligatory contribution of manufacturers for reimbursed pharmaceuticals are as follows (these conditions are determined in the Act XCVIII of 2006):

- 20% of the reimbursement amount calculated on ex-factory price level as payback for all reimbursed drugs,
- 10% tax for innovative drugs with a history of at least 6 years of reimbursement and without reimbursed generic alternatives.

Additionally, central tenders are used as itemized reimbursed technique for high-priced medicines^22^. There are also prescribing quotas for physicians to prescribe a predefined percentage of preferred reference product within their prescription.

In Latvia, managed entry agreements are allowed by legislation. RSAs are used in a form of simple discounts, price-volume agreements and payback. In some cases pay-per-performance are used.

In Lithuania, RSAs, pay-for-performance agreements and fixed volume agreements are used to optimize expenditure for costly medicines. Public tendering is obligatory for purchases of medicines for hospitals.

In Poland, fixed price is used in the case of ambulatory drugs (no tendering) but in the case of reimbursed ambulatory drugs the maximal price is used (Kawalec and Malinowski, 2016; Jahnz-Rózyk et al., 2017). Public tendering is obligatory for purchases of medicines for public hospitals. On retail market, any rebates are prohibited (fixed pricing) but in the case of hospitals rebates or discounts are allowed.

In Romania, cost-volume contracts and cost-volume-outcome contracts are applied. Pure RSAs are not available in reimbursement decision making, but a clawback tax is applied, that is, manufactures of drugs must return some of the profits made from sales of free/compensated drugs.

In Slovakia, there are special assessment criteria for highly innovative medicines which have no treatment alternatives (including orphan drugs)—the threshold value used in cost-effectiveness analysis is not applicable for this type of drugs. The reimbursement decision is made after negotiations between MAH and health insurance companies. Usually, price discounts or price limits are offered for public reimbursement and RSAs are rarely used in Slovakia.

### A review of health technology assessment in CEE region countries

Czech Republic, Estonia, Latvia, Lithuania and Slovakia require the same set of HTA documentation, including systematic review, pharmacoeconomic analysis, and BIA. In Hungary, additional data is required, such as drug prices review in reference countries, indication and targeted reimbursement level, and pharmacoeconomic studies from other countries^22^ (see Tables 7, 8 for details).

**Table 7:** Health Technology Assessment in considered CEE countries.

**Aspect required**	**Bulgaria**	**Croatia**	**Czechia**	**Estonia**	**Hungary**	**Latvia**	**Lithuania**	**Poland**	**Romania**	**Slovakia**
Is HTA dossier obligatory in pricing and reimbursement submission?	Yes	No but alternative assessment has to be submitted	Yes	Yes	Yes	Yes	Yes	Yes	Yes	Yes
In case if HTA is not obligatory it could be submitted with a reimbursement application as a additional facultative dossier?	It's obligatory	Yes	It's obligatory	It's obligatory	It's obligatory	It's obligatory	It's obligatory	It's obligatory	It's obligatory	It's obligatory
Are HTA guidelines obligatory for submitted HTA dossier attached to the reimbursement application?	Yes	Yes	Yes	Yes	Yes	Yes	Yes	Yes	Yes	Yes
What institutions are responsible for determining “benefit” and “value” of pharmaceuticals?	HTA Commission National Council Ministry of Health	The CHIF and their Expert council of the CHIF	State Institute for Drug Control (SUKL)	Ministry of Social Affairs Health Insurance Fund	The National Institute of Health Insurance Fund Management (since 2016) The HTA Office at the National Institute of Pharmacy and Nutrition The Medical Professional Colleges The Technology Assessment Committee	The National Health Service of Latvia	Ministry of Health The State Medicines Control Agency National Health Insurance Fund under the Ministry of Health	Polish HTA Agency (Agency for Health Technology Assessment and Tariff Systems; AOTMiT)	National Agency for Medicines and Medical Devices in Romania	Slovak Ministry of Health The Reimbursement Committee

**Table 8:** Obligatory parts of HTA dossier in considered CEE countries.

**Type of analysis**	**Bulgaria**	**Croatia**	**Czechia**	**Estonia**	**Hungary**	**Latvia**	**Lithuania**	**Poland**	**Romania**	**Slovakia**
Clinical analysis	+	−	+	+	+	+	+	+	−	+
Pharmacoeconomic analysis (cost effectiveness cost utility or cost minimalization)	+	−	+	+	+	+	+	+	−	+
Budget impact analysis	+	+	+	+	+	+	+	+	+ A simple budget impact model (only direct costs)	+
Other analyses used in reimbursement application process	Ethical considerations	−	−	−	Drug price in other countries assessment, requested indication and targeted reimbursement technique/level, pharmacoeconomic studies from other countries	−	−	Decision problem analysis, rationalization analysis	HTA from France, Germany and UK, number of EU countries with a positive reimbursement status, real-world data (RWD) study	−

In Bulgaria, a full HTA is performed for medicinal products belonging to a new INN group, which is not included in the Positive Drug List; HTA dossier could also be submitted for a new pharmaceutical form, new dosage form, and for biosimilars. Minister of Health issued HTA guidelines in a form of a Regulation, and all submitted HTA dossier must fulfill them. The HTA Commission, responsible for conducting the assessment of health technologies, is a group of representatives of: National Council, Ministry of Health, Bulgarian Drug Agency, National Health Insurance Fund, and National Centre for Public Health. The National Council for Pricing and Reimbursement of Medicinal Products is responsible for pricing and for inclusion of medicinal product in PDL. It is a state commission, and members are elected by the Council of Ministers.

According to the Act No. 48/1997, HTA process in Czech Republic is an obligatory part of reimbursement decision-making process. HTA guidelines, according to which SUKL makes assessment of HTA dossiers, are publicly available and compliance with these guidelines is highly recommended^23^.

In Croatia, the criteria for inclusion of medicines in the reimbursement list are as follows: the importance of the drug from the standpoint of public health, the therapeutic importance of the drug, relative therapeutic value of the drug, and the assessment of the ethical aspects^9^^,^^10^. HTA dossier could be submitted with a reimbursement application as an additional, facultative dossier, but the Budget Impact Analysis is obligatory for submission, while systematic review/clinical analysis and pharmacoeconomic analysis are not obligatory (except in specific cases). It is required that BIA contains 2 scenarios of the analytical model, where one takes into account only the direct costs of the drug and the other contains all the additional direct costs arising from placing the drug on the reimbursement list. MAHs submit the proposal for inclusion of a new drug on the reimbursement list or the expansion of indications of the already included drug. Guidelines for the BIA issued by the CHIF include basic methodology requirements for BIAs and are in accordance with the International Society for Pharmacoeconomics and Outcomes Research (ISPOR) guidelines (Weinstein et al., 2003; Mauskopf et al., 2007).

The Agency for Quality and Accreditation in Health Care is responsible for the HTA process in Croatia. The activity of the agency contains national collaboration, education, and the HTA promotion, international collaboration (EU Projects), the establishment of the Croatian HTA Guideline, and the creation of the HTA reports and scientific publications (Voncina and Strizrep, 2010; Vogler et al., 2011). The assessment process starts with a pre-assessment of the existing evidence on each selected topic, prepared by the HTA Department. It is intended that the HTA Agency will take part in future pricing and reimbursement decisions because currently its activity is still on the level of recommendation.

In Lithuania, the national HTA agency has not been established till now. Assessment of dossier for reimbursement is performed by three institutions: State Medicines Control Agency—therapeutic value, Department of Pharmacy of Ministry of Health—pharmacoeconomic value, and National Health Insurance Fund (NHIF)—budget impact assessment. Therapeutic value of the product assessment is based on the innovativeness (2–5 points) and therapeutic benefit for patients (3–10 points). Pharmacoeconomic value score is a composite of relative pharmacieconomic value and declared price scores, and may range from 1.5 to 7.5.

Recommendations on reimbursement are issued by the Committee of reimbursement, and are following:

- the medicine is recommended to be included to positive list A if therapeutic value is ≥ 9, pharmacoeconomic value ≥ 4, and there is negative (neutral) impact on NHIF budget;
- the medicine is recommended to be included to the waiting list, if therapeutic value is ≥ 11, pharmacoeconomic value ≥ 4, and there is positive impact on NHIF budget;
- the medicine is not recommended for reimbursement, if therapeutic value is < 9, pharmacoeconomic value < 4.

Waiting list is revised every half a year, and medicines are included to the Positive list if payers budget is able to afford it. Recommendation of reimbursement committee goes to Obligatory Health Insurance Council, which issues recommendation to the Minister of Health.

In Poland, HTA is obligatory in the application process for reimbursement. The full HTA report has 3 basic components: clinical analysis, economic analysis, and BIA. The clinical analysis evaluates the clinical efficacy and safety profile. The economic analysis informs whether the use of a new, reimbursed drug in place of standard therapy is cost-effective. In other words, the analysis has to determine whether the marginal cost per quality-adjusted life-years gained is below the cost-effectiveness threshold of 130,000 PLN (3 × Gross Domestic Product per capita). Finally, the BIA assesses the financial burden of a reimbursement decision for the National Health Fund. Rationalization analysis is performed only if the BIA revealed additional costs for public payer resulted from the reimbursement of assessed technology; this analysis has to provide proposals of rationalization solutions, implementation of which should generate savings in an amount equaled at least the value of additional costs revealed in the BIA^24^.

Reimbursement application should be submitted to the Ministry of Health with HTA dossier. Based on the submitted dossier, the President of the AHTAPol issues 3 types of recommendations. The first type is positive recommendation, which supports coverage from the public budget. The second type is conditional—the reimbursement is possible but additional conditions have to be met (e.g., price reduction, RSA). A negative recommendation advocates lack of coverage from public resources. After the AHTAPol issues its recommendation, negotiations with the Economic Committee of the Ministry of Health (official price terms of reimbursement) are crucial for reaching a final decision on reimbursement (Kawalec and Malinowski, 2016)^24^.

In Romania, HTA dossier is obligatory in reimbursement submission for a new molecules, compensated molecules for indication expansion, and generics or biosimilars that do not have compensated molecules in the list. The scorecard HTA model was established in 2014 and it was based on the analysis of 6 criteria: HTA decisions from France, Germany, and UK, the number of EU countries (27 countries) in which the drug is reimbursed, and a BIA focusing on direct costs. A full HTA dossier can be submitted with a reimbursement application as additional facultative dossier^25^.

Romanian HTA guidelines in its current version make a comprehensive evaluation of the technology impossible because they take into account only cost-saving aspect and ignore the efficacy and safety (Radu et al., 2016). There is a proposed transition to a model that also includes studies of cost-effectiveness and calibration of utility values specific to Romania (Paveliu, 2013).

In Slovakia, full HTA dossier is not obligatory in pricing and reimbursement submission for generics and biosimilars. However, for innovative drugs clinical assessment review, pharmacoeconomic analysis, and BIA are required. Slovak Ministry of Health established the Reimbursement (or Categorization) Committee to act as its advisory body in reimbursement processes. The Categorization Committee constitutes of 3 representatives from the Ministry of Health, 5 representatives from health insurance companies and 3 representatives from the Slovak Medical Chamber. The Categorization Committee is supported by different advisory working groups, medical board (assessing the effectiveness, safety, and importance of the medicine) and the Working Group for Pharmacoeconomics, Clinical Outcomes and Health Technology Assessment of the Ministry of Health. Recommendations from the Categorization Committee are the basis for the Ministry of Health when issues the final decisions (details in Table 9).

**Table 9:** Determinants for therapeutic and social value of pharmaceuticals in Slovakia.

**Determinants for the therapeutic value of drugs**	**Determinants for the social value of drugs**
Effectiveness	Severity of the disease
Safety	Impact on society if not treated (e.g., spread of infection)
Cost-effectiveness	Social value (e.g., orphan drugs)
A first or second option or adjunctive treatment	Risk of abuse
Causal treatment, prophylaxis or a symptomatic treatment	Impact on total costs

## Discussion

Comparative analysis, which included 10 countries, confirmed that the pricing and reimbursement requirements are quite similar among the countries of the CEE region. However, some differences were also identified.

Within this analysis, the expenditures on reimbursement were also assessed and expressed in relation to the total expenditure on health. Among the compared countries, the relation of drug reimbursement costs to total public health care spending was from 0.12 to 0.21, with a median value between 0.15 and 0.18, depending on the analyzed year.

In all analyzed countries the reference price system, including external reference pricing and internal reference pricing, is used. External reference pricing consists in comparing the prices of the same product in other countries. Particular countries refer to different number of countries—from 3 (Estonia, Switzerland) to as much as 31 (Poland). Slovakia is the most frequently referenced country, while Cyprus, Iceland, Malta, Luxembourg, and Norway are countries to which other CEE countries refer least frequently. Internal reference pricing is meant to determine pharmaceutical prices based on similar products or drug equivalents available on pharmaceutical market. It is used to set reimbursement prices for product groups called clusters. Pharmaceuticals are clustered according to active substance; several active substances are clustered together if they are chemically related and pharmacologically equivalent (Panteli et al., 2016). Value-based pricing has been gaining importance as an alternative strategy in recent years (Panteli et al., 2016) and some elements of it are applied in Poland and Hungary.

Considered countries use publicly available price information which does not present confidential rebates and discounts negotiated between manufacturers and payers (Panteli et al., 2016). In all of the countries, reimbursement decision-making processes in the outpatient sector refer to a PDL (Panteli et al., 2016); pharmaceuticals are reimbursed once they are included in the list, except in Bulgaria where new INNs start to be reimbursed at the beginning of the year after their inclusion. Some countries apply both a positive and a negative drug list, although the negative drug list is rare and is present only in 3 countries (Hungary, Romania and Slovakia). Reimbursement decisions are regularly revised and updated in almost all of included countries (except for Hungary and Lithuania), most commonly using a 2–12-month window. *Ad hoc* revisions are additionally carried out following changes or extensions of an indication, availability of new evidence or market entry of a therapeutic alternative (Panteli et al., 2016). *Ad hoc* revisions are made in Hungary and Lithuania and they are also possible in Czech Republic and Romania as an alternative to the periodic revisions.

RSAs become more and more popular in Europe (Panteli et al., 2016), and they are currently available in all analyzed CEE countries. The situation looks different in the case of cap for patients, as they are only used in Estonia and Romania. Cap for patients are also possible in Slovakia and Bulgaria but only for the vulnerable groups of patients. Specific discounts are sometimes used for particularly costly pharmaceuticals. While such discounts are agreed, rebates are returned to the payer after the purchase of a pharmaceutical. Discounts and rebates can be applied universally (legally imposed and pertaining to all manufacturers and payers in the system) or be negotiated between individual payers and manufacturers. In the case of inpatient care, tendering can be employed.

RSAs as informal and confidential agreements influence drug availability in considered countries as they usually significantly decrease costs of therapy thus make it possible to reimburse it for patients. Due to lack of relevant cost data the difference between official prices and real costs of medicines is a substantial information gap. Without knowing the figures the possible effects of RSAs on the national drug policies were very difficult for evaluation; we could only presume that their influence on disturbing the transparency of pricing policy could be significant.

Cost sharing usually applies to pharmaceuticals prescribed in outpatient care and most commonly takes the form of a percentage share of the retail price of a medicine. The amount of this share can vary depending on the condition (e.g., chronic diseases), income or employment status, or age. Copayments for outpatient care are common and present in all analyzed countries, which determine the price share to be borne by the statutory health system as part of the reimbursement decision for each medicine. The available levels of reimbursement differ within and between the countries and range from 20 to 100%.

According to our survey only 3 countries in the sample (Czech Republic, Lithuania, and Slovakia) use additional arrangements to enable better access to orphan drugs. In Czech Republic, orphan drugs sometimes meet the criteria for highly innovative medicines and get temporary reimbursement up to 3 years even without relevant cost-effectiveness and BIA. In Slovakia special reimbursement regulations are used in case of orphan drugs (with prevalence lower than 1: 100,000)—the threshold value used within the cost-effectiveness analysis is not applicable for this group of drugs.

Generic substitution is also possible in almost all analyzed CEE countries, while some make it mandatory (Estonia, Hungary if available (this is mainly a generic product from interchangeability list, with the same dose, INN and drug form). Bulgaria is the only country in which pharmacists cannot make any changes on the reimbursable prescriptions, because the coding system use and the details of the MAH, regardless of whether it is an originator or a generic. In Romania, the generic substitution is also mandatory, but with some exceptions as biologic drugs cannot be substituted. To support generic substitution, prescription of active substance (INN) rather than trade name has been institutionalized in all countries—in some countries it is mandatory (Estonia, Lithuania, partially Romania, and Slovakia), while in others it is optional. Implementation of relevant regulation also influences the market penetration of generic products. In terms of volume, the share of generics in the analyzed CEE countries was on average 60% (median 69%) and in terms of value it was 33% (median 30%). In terms of volume, Bulgaria (78%) (Stoimenova et al., 2016), Latvia (77%), and Romania (75%) take the top 3 places in the sample, while in terms of value, it was Latvia (59%), Bulgaria (45%), and Poland (41%). The lowest share of generics was observed in Estonia (36% in terms of volume and 16% in terms of value).

To increase the access to a drug and bring more stability in reimbursement and pricing system in Romania, in 2016, the government issued a decision approving the methodology regarding the calculation method and the procedure for endorsement and approval of the maximum prices; the new methodology will reduce the price of the drugs.

Drugs for compassionate use are reimbursed only in Hungary and Czech Republic; special early drug access programs tend to facilitate the availability of new medicines to patients suffering with life threatening diseases and provide them an access to drugs not available via standard reimbursement. In Hungary, the vulnerable patients with very low income are granted a monthly individual budget for their essential prescription-only medicines. In Czech Republic, drugs which are not authorized may be reimbursed under specific conditions, and additionally the individual reimbursement is also possible (specific product for a specific patient after acceptance of individual application by the payer).

Electronic prescribing is available in some of the countries. In Bulgaria, Hungary, Poland, Czech Republic, and Slovakia, the implementation of electronic prescribing is planned.

HTA is carried out in all analyzed CEE countries and HTA dossier is obligatory in pricing and reimbursement submission. The only exception is Croatia, where alternative assessment has to be submitted instead of a HTA dossier. The key analyses included in HTA dossier are: clinical analysis (obligatory in 8 out of 10 countries), pharmacoeconomic analysis (obligatory in 8 out of 10 countries), and BIA (obligatory in all countries). Other additional parts of HTA dossier which are obligatory only in some of the considered countries are: ethical considerations (Bulgaria), submitting drug price in other countries (Hungary), submitting published pharmacoeconomic analysis or heath technology assessments from other countries (Hungary, Romania), decision problem analysis (Poland), rationalization analysis (Poland).

In all considered countries specific committees responsible for formulating reimbursement recommendations have been established. Criteria guiding recommendations as well as final reimbursement decision-making vary among the countries in this study. Slovakia has its unique criteria determining the therapeutic and social value of medicines. It can be assumed that the therapeutic value is determined in a similar way in all other countries by considering the following aspects: effectiveness, safety, cost-effectiveness, whether it is a causal treatment, prophylaxis, or a symptomatic treatment, whether it is a first or second option or adjunctive treatment. The following aspects are taken into account in Slovakia while considering social value of a medicine: severity of the disease, impact on society if not treated, social value (e.g., orphan drugs), risk of abuse, impact on total costs.

The national HTA assessment institutions or authorities are in some cases also responsible for the final political decision on reimbursement and/or marketing authorization. In some cases, for example in Poland, final decisions or their implementation can differ from the reimbursement recommendations, usually as a result of budgetary or societal considerations (Kawalec and Malinowski, 2016).

The current project is quite innovative as we did not identify any other studies on the similar topic, carried out for CEE countries. However, some similar reports were found.

In another publication the Hungarian reimbursement system was presented (Endrei et al., 2014). In this country, there was a solidarity-based health insurance system with a single payer, and the National Institute of Health Insurance Fund Management was the only health care financing agency. The Hungarian HTA office belonged to the National Institute of Pharmacy and Nutrition (Országos Gyógyszerészeti és Élelmezés-egézségügyi Intézet). Hungary adopted the directive on transparency for the evaluation of new drugs and medical devices applying for health insurance reimbursement. There were also formal guidelines for conducting economic evaluation of health care interventions.

Gulacsi et al. (2014) described and discussed the development and use of HTA in 5 CEE countries: Bulgaria, Czech Republic, Hungary, Poland, and Romania. The study was based on document analysis and expert opinion; the similarities and differences between the particular CEE countries in HTA were presented. In 4 of the discussed countries, that is, Hungary, Poland, Romania, and Bulgaria, HTA was embedded in the law. The Czech Republic had legal embedding of HTA, cost-effectiveness analysis, and BIA for the purposes of evaluating therapies. Each of the countries had a HTA body which played a role in reimbursement decision-making, although their competences varied. Most bodies produced reimbursement recommendations based on an evaluation of dossiers provided by pharmaceutical companies rather than in-depth assessments. In all countries, except Czech Republic, the final reimbursement decisions belonged to the ministry of health. Guidelines for HTA were different in terms of detailed methodology required but the evaluation of the following issues was generally required: information on clinical efficacy and safety, systematic reviews, meta-analysis, epidemiology, results from health economics analysis, disease burden, and patient-reported outcomes. There were differences in the criteria for positive recommendations in the analyzed countries related to safety issues, implementation of financing thresholds, and the influence of reimbursement status in other countries.

The time elapsed from marketing authorization to the starting date of reimbursement of the original medicines in CEE countries (Austria, Slovakia, Slovenia, Bulgaria, the Czech Republic, Latvia, Lithuania, Estonia, Hungary, Poland, and Romania) was analyzed in the study by Komáromi et al. (2014). The analysis included 216 products and the following indicators were calculated: the delay between marketing authorization date and reimbursement date, the number of reimbursed INNs according to a specific country or MAH, success rate as the ratio of reimbursed INNs to examined INNs. The average number of days which elapsed between the authorization and the starting date of reimbursement differed between the analyzed countries—it ranged from 403 days in Slovenia to 1,295 days in Poland. The average value in all included countries was 632 days.

A recent study compared the prices and reimbursement of cardiovascular medicines in Bulgaria and Romania. The results showed that the lower margins in Bulgaria may compensate the higher value-added tax and can lead to lower retail price (Mitkova et al., 2016).

In the study (Oyebode et al., 2015), all of the specific requirements for the HTA dossier in individual European national agencies responsible for reimbursement were collected, analyzed, and compared. Twenty-nine countries were analyzed, including some countries from the CEE region. The level of detail of the required information varied considerably across countries. Despite differences in quantity and detail, the content of the evidence requirements was rather similar and the following points were required: information on target disease, safety and clinical effectiveness of considered pharmacotherapy.

## Conclusions

Pricing and reimbursement requirements are quite similar across the CEE region, although some differences were identified. External reference pricing as well as internal reference pricing is common in all analyzed countries. Positive reimbursement lists are valid in all countries, and negative ones are rarely used. Reimbursement decisions are regularly revised and updated in the majority of countries. Copayment is common and available levels of reimbursement differ within and between the CEE countries and range from 20 to 100%. Risk-sharing schemes are often in use, especially in the case of innovative, expensive drugs. Generic substitution is also possible in the analyzed CEE countries, while some make it mandatory. HTA is carried out in the majority of CEE countries and HTA dossier is obligatory in pricing and reimbursement submission.

## Author contributions

PK conceived the conception and design of the study, including protocol and questionnaires preparation; PK coordinated the project. PK contributed in acquisition of data and data management. PK and ES carried out statistical analysis, interpretation of data and prepared the draft of the manuscript. TT, LV, PD, MM, AS, GP, ZR, AM, CS, AH, AI, AT-S, JG collected and provided input data for corresponding countries. All authors contributed to editing the manuscript and approved the final version submitted for publication. PK is the guarantor.

### Conflict of interest statement

The authors declare that the research was conducted in the absence of any commercial or financial relationships that could be construed as a potential conflict of interest.

## Footnotes

^1^http://www.ema.europa.eu/ema/index.jsp?curl=pages/regulation/general/general_content_001595.jsp&mid=WC0b01ac0580b18a3d (Accessed on June 02, 2017)

^2^https://ec.europa.eu/growth/sectors/healthcare/competitiveness/products-pricing-reimbursement/transparency-directive_en (Accessed on June 02, 2017)

^3^Council Directive 89/105/EEC of 21 December 1988 relating to the transparency of measures regulating the prices of medicinal products for human use and their inclusion in the scope of national health insurance systems. http://eur-lex.europa.eu/legal-content/EN/TXT/?uri=CELEX:31989L0105 (Accessed on June 02, 2017)

^4^https://ec.europa.eu/neighbourhood-enlargement/policy/from-6-to-28-members_en (Accessed on June 02, 2017)

^5^Act XCVIII of 2006 on the Safe and Economic Supply and Distribution of Medicines and Therapeutic Medical Devices. 2006; [cited 4 April 2017]. https://net.jogtar.hu/jr/gen/hjegy_doc.cgi?docid=A0600098.TV (Accessed on June 02, 2017)

^6^Regulation of Ministry of Social Affairs: Procedure for drafting and amendment of a list of medicinal products of the Estonian Health Insurance Fund and the content of criteria for establishing the list and evaluators of compliance with the criteria. https://www.riigiteataja.ee/en/eli/ee/SOM/reg/516062015002/consolide (Accessed on June 02, 2017)

^7^Order of Minister of Health of Lithuania Nr., 159 (5 April 2002) https://www.e-tar.lt/portal/lt/legalAct/TAR.E83CFAB10E7B (Accessed on June 02, 2017)

^8^Ministry of Health of the Republic of Bulgaria and the International Bank for Reconstruction and Development, Final Report on Health Financing Diagnostic and Review of Envisaged Reforms, May 2015.

^9^Medicinal Products Act (“Official Gazette”, no. 76/2013) http://www.zakon.hr/z/399/Zakon-o-lijekovima (Accessed on June 02, 2017).

^10^The Ordinance on the criteria for marketing authorization of drugs on the Basic and Additional drug list of CHIF, http://www.hzzo.hr/zdravstveni-sustav-rh/pravilnik-o-mjerilima-za-stavljanje-lijekova-na-osnovnu-i-dopunsku-listu/ (Accessed on June 02, 2017).

^11^Act No. 48/1997, on Public Health Insurance, Czech Republic.

^12^Decree 32/2004 (26 April) of the Ministry of Health, Social and Family Affairs. http://net.jogtar.hu/jr/gen/hjegy_doc.cgi?docid=A0400032.ESC (Accessed on June 02, 2017).

^13^Resolution of the Government of the Republic of Lithuania, https://www.e-tar.lt/portal/lt/legalAct/TAR.9E67C6BA5E0E (Accessed on June 02, 2017).

^14^Emergency Government Ordinance no. 59/28.09.2016 regarding amendment of Law no. 95/2006 on healthcare reform, Romanian Official Gazette 768/ September 30, 2016.

^15^Government Decision no. 800/26.10.2016 October 31, 2016 approving the methodology regarding the calculation method and procedure for endorsement and approval of the maximum prices of drugs in Romania, Romanian Official Gazette 869/October 31, 2016.

^16^Act No. 363/2011 Coll. on the scope and conditions of payments for medicines, medical devices and dietetic foods from public health insurance and amending certain acts, as amended. http://www.zakonypreludi.sk/zz/2011-363 (Accessed on June 02, 2017).

^17^http://www.sukl.eu/sukl/list-of-reimbursed-medicinal-products-1., (Accessed on June 02, 2017).

^18^http://www.sukl.cz/sukl/seznam-leciv-nehrazenych-ze-zdravotniho-pojisteni., (Accessed on June 02, 2017).

^19^Regulation of Ministry of Social Affairs in Estonia: Co-payment rates. https://www.riigiteataja.ee/akt/128092012015 (Accessed on June 02, 2017)~Regulation of Ministry of Social Affairs in Estonia: Co-payment rates. https://www.riigiteataja.ee/akt/128092012015 (Accessed on June 02, 2017).

^20^Health Insurance Act; Estonia https://www.riigiteataja.ee/en/eli/ee/Riigikogu/act/529122016002/consolide (Accessed on June 02, 2017).

^21^Positive list, Latvia. http://www.vmnvd.gov.lv/lv/kompensejamie-medikamenti/kompensejamo-zalu-saraksts (Accessed on June 02, 2017).

^22^Decree No. 9/1993. (IV. 2.) NM of the Minister of Welfare on the Social Insurance Financing of Specialist Services [cited 4 April 2017]. https://net.jogtar.hu/jr/gen/hjegy_doc.cgi?docid=99300009.nm. (Accessed on June 02, 2017).

^23^http://www.sukl.cz/leciva/metodiky-stanoveni-cen-a-uhrad., (Accessed on June 02, 2017).

^24^http://www.aotm.gov.pl/www/, January 2016 (Accessed on June 02, 2017)

^25^Ministry of Health Order No. 487/2017 regarding the approval of criteria for evaluation of medical technologies, the documents to be submitted by solicitants, the process and the methodology used to assess the drugs, the new indications to be included on the reimbursement list, and the criteria for exclusion of drugs from the list. Romanian Official Gazette June 28th, 2017.
